# CAY10683 and imatinib have synergistic effects in overcoming imatinib resistance *via* HDAC2 inhibition in chronic myeloid leukemia

**DOI:** 10.1039/c9ra07971h

**Published:** 2020-01-03

**Authors:** Tianzhuo Zhang, Danna Wei, Tingting Lu, Dan Ma, Kunlin Yu, Qin Fang, Zhaoyuan Zhang, Weili Wang, Jishi Wang

**Affiliations:** Department of Clinical Medical School, Guizhou Medical University Guiyang 550004 PR China; Department of Hematology, Affiliated Hospital of Guizhou Medical University Guiyang 550004 PR China +86 851 675 7898 +86 136 390 89646 wangjishi9646@163.com; Department of Guizhou Province Hematopoietic Stem Cell Transplantation Center, Key Laboratory of Hematological Disease Diagnostic and Treatment Centre Guiyang 550004 PR China; Department of Hematology and Oncology, Guiyang Maternal and Child Health Hospital Guiyang 550002 PR China; Department of Pharmacy, Affiliated Hospital of Guizhou Medical University Guiyang 550004 PR China

## Abstract

Imatinib (IM) is utilized for targeting the BCR–ABL fusion protein and as such, chronic myeloid leukemia (CML) is considered to be a curable disorder for which patients can achieve a long survival. However, 15–20% CML cases end up with IM resistance that will develop into the accelerated stage and eventually the blast crisis, thereby restricting the treatment choices and giving rise to a dismal survival rate. Histone deacetylases (HDACs) have been identified to modulate the oncogene as well as tumor suppressor gene activities, and they play crucial parts in tumorigenesis. It is found recently that IM combined with HDAC inhibitors (HDACi) can serve as a promising means of overcoming IM resistance in CML cases. Santacruzamate A (CAY10683) has been developed as one of the selective and powerful HDACi to resist HDAC2. Therefore, in this study, we aimed to examine whether CAY10683 combined with IM could serve as the candidate antitumor treatment for CML cases with IM resistance. The influences of CAY10683 combined with IM on the cell cycle arrest, apoptosis, and viability of CML cells with IM resistance were investigated, and it was discovered that the combined treatment exerted synergistic effects on managing the IM resistance. Moreover, further studies indicated that CAY10683 combined with IM mainly exerted synergistic effects through inhibiting HDAC2 in K562-R and LAMA84-R cells with IM resistance. Besides, the PI3K/Akt signal transduction pathway was found to mediate the HDAC2 regulation of CML cells with IM resistance. Eventually, it was also discovered, based on the xenograft mouse model, that the combined treatment dramatically suppressed CML proliferation *in vivo*. To sum up, findings in the current study indicate that CAY10683 combined with IM can be potentially used as the candidate treatment for CML with IM resistance.

## Abstract 2

Imatinib (IM) is utilized for targeting the BCR–ABL fusion protein and as such, chronic myeloid leukemia (CML) is considered to be a curable disorder for which patients can achieve a long survival.

## Graphical abstract

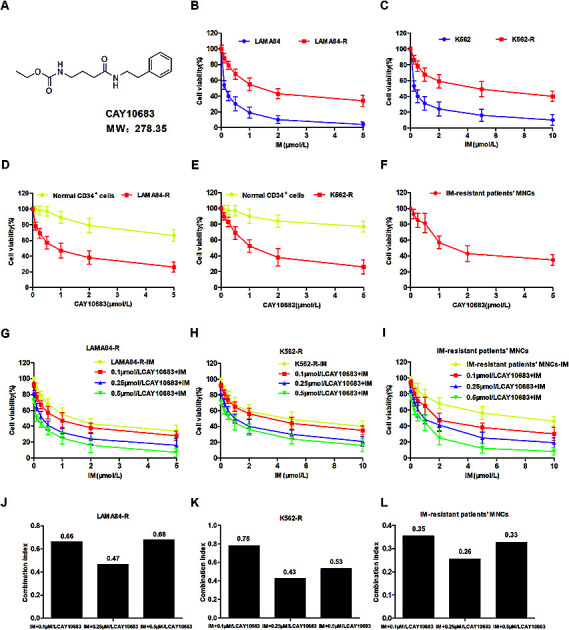

## 1 Introduction

Chronic myeloid leukemia (CML) has been identified as a hematologic disorder, which features the reciprocal translocation between the Abelson leukemia virus oncogene (ABL) localized in chromosome 9 (also named the Philadelphia (Ph) chromosome) and the break-point cluster (BCR) gene localized in chromosome 22. Typically, the fusion protein BCR–ABL can activate the activity of tyrosine kinase constitutively and induce a variety of downstream signal transduction pathways like the mitogen-activated protein kinase (MAPK) and the Akt pathways, which can thereby facilitate the proliferation of cells and suppress the apoptosis of cells, leading to genetic instability.^1^ After the discovery of efficient tyrosine kinase inhibitors (TKIs) like imatinib (Gleevec or Glivec) together with the derivatives nilotinib, dasatinib, ponatinib and bosutinib, CML became a curable chronic disorder that achieved long-time survival of over 85%.^2^ Typically, the inhibiting mechanisms for imatinib (IM) mesylate include preventing the activation and phosphorylation of substrate from BCR–ABL, and binding to the ATP-binding pocket in BCR–ABL. However, IM resistance has become a leading obstacle in clinical CML treatment, which may be ascribed to the mutations and over-expression of BCR–ABL, as well as some BCR–ABL-independent pathways. As a result, about 15–20% of CML cases will finally have IM resistance and develop to the accelerated stage and ultimately the blast crisis.^3^ Therefore, it has become a research hotspot to overcome the IM resistance in CML treatment. Recently, it was suggested that the IM resistance in CML cells should be handled through the combined treatment of BCR–ABL kinase inhibitor with histone deacetylase (HDAC) inhibitors.^4–6^

Histone deacetylase (HDAC) mediates non-histone and histone deacetylation and regulates transcriptional factor activities participating in tumor progression and initiation, and in regulating many critical proteins (like tumor suppressor gene) at the post-transcription level.^7,8^ The HDAC inhibitors (HDACi) are promising for use in treating some cancer types.^9^ The Food and Drug Administration (FDA) has approved the clinical use of five HDACi for bipolar disorders, multiple myeloma, and peripheral T-cell lymphoma.^10^ Additionally, many clinical studies are carried out using HDACi alone or combined with traditional or new chemotherapeutics to treat cancers.^11^ For example, Wang *et al.* suggested that divalproex sodium, an HDACi, promotes the IM effect against leukemia within the cells of chronic myeloid leukemia, which is partially achieved by SIRT1.^12^ Therefore, HDACi combined with IM may serve as a promising treatment to overcome the resistance to IM of CML.

Santacruzamate A (CAY10683) is a natural product obtained from the Panamanian marine cyanobacterium that is reported to be a selective and powerful HDACi for HDAC2.^13^ CAY10683 has obtained clinical approval due to its immunomodulatory and antiproliferative effects,^13^ and is consequently utilized for treating breast cancer^14^ and cutaneous T-cell lymphoma.^15^ So far, no study has been carried out to examine the role of CAY10683 in CML resistant to IM, and the effects of CAY10683 combined with IM on CML cells resistant to IM remain unclear.

According to many studies, the over-expression of HDAC is a distinct mechanism of leukemia resistance.^16,17^ As one of the Class I HDACs members, HDAC2 plays a vital role in hematopoietic development and cell proliferation.^18,19^ According to our previous study, HDAC2 shows higher expression in K562-R cells resistant to IM as compared to K562 cells sensitive to IM, indicating that HDAC2 may exert a critical role in IM resistance.^20^ The findings of the current study suggest that IM combined with CAY10683 (a selective HDAC2 inhibitor) exert synergy mainly by inhibiting HDAC2 in K562-R and LAMA84-R cells resistant to IM. Moreover, the PI3K/Akt signal transduction pathway modulated HDAC2 regulation in CML cells resistant to IM. Eventually, it was discovered through the xenograft mouse model that the combined treatment remarkably suppressed CML proliferation *in vivo*. The above experiments provide a promising treatment for handling CML resistance to IM, and the current study is the first to report a treatment that combines IM with CAY10683, as well as its synergy in treating CML resistant to IM.

## 2 Materials and methods

### 2.1 Cell lines and culture

Two IM-sensitive CML cell lines, LAMA84 and K562, as well as their resistant counterparts LAMA84-R and K562-R cell lines were obtained from the center laboratory of the Hematopoietic Stem Cell Transplantation Center of Guizhou Province (Guiyang, China). LAMA84-R and K562-R cells were maintained in RPMI 1640 medium with IM concentration increasing (0.1, 0.2, 0.3 and 0.5 μM) every 2 weeks of culture. The final resistance of LAMA84-R cells to IM was 11.03-fold that of LAMA84 cells. Similarly, the final resistance of K562-R cells to IM was 16.02-fold that of K562 cells. The cell lines were cultured in RPMI-1640 medium (Gibco, Carlsbad, CA, USA) supplemented with 10% fetal bovine serum (Tianhang Biotechnology, Zhejiang, China) and 1% penicillin/streptomycin (Invitrogen, Carlsbad, USA) at 37 °C/5% CO_2_.

### 2.2 Ethical statement

Informed consent was signed by all human subjects. This study was performed in strict accordance with the NIH guidelines for the care and use of laboratory animals (NIH publication no. 85-23 rev. 1985) and was approved by the Biomedical Ethics Committee of Affiliated Hospital of Guizhou Medical University (Guiyang, China).

### 2.3 CD34^+^ cell isolation

Normal CD34^+^ cells were isolated from mobilized healthy donors by using the MASC human CD34 microbead kit according to instructions. Isolated mononuclear cells (MNCs) were cultured in DMEM: F12 medium containing 10% FBS in the presence or absence of GM-CSF (100 ng mL^−1^). Isolated CD34^+^ cells were cultured in serum-free HSC expansion medium. All primary cells were cultured at 37 °C for up to 7 d.

### 2.4 Patient samples

A total of 3 patients who were diagnosed with CML and received standard IM therapy at the Affiliated Hospital of Guizhou Medical University were included in the study after written informed consent was obtained. Peripheral blood samples were collected from healthy donors and the patients who were diagnosed using the WHO classification in Table 3. The current study was approved by the Institutional Review Board of the Affiliated Hospital of Guizhou Medical University.

**Table 1:** Sensitivity of imatinib-sensitive and resistant cell lines to imatinib, CAY10683 or their combination^a^

Cell lines	IM + CAY10683 (μM)	IC_50_ value (μM)
LAMA84	IM	0.1411 ± 0.03
LAMA84-R	IM	1.5560 ± 0.33
	CAY10683	0.8579 ± 0.07
	IM + 0.1 CAY10683	0.8523 ± 0.05*
	IM + 0.25 CAY10683	0.2749 ± 0.01**
	IM + 0.5 CAY10683	0.1460 ± 0.07**

a The results are expressed as the mean ± SEM. *n* = 3. *P < 0.05 and **P < 0.01 *versus* the IM group.

**Table 2:** Sensitivity of imatinib-sensitive and resistant cell lines to imatinib, CAY10683 or their combination^a^

Cell lines	IM + CAY10683 (μM)	IC_50_ value (μM)
K562	IM	0.2847 ± 0.05
K562-R	IM	4.5610 ± 1.06
	CAY10683	1.2630 ± 0.21
	IM + 0.1 CAY10683	3.2025 ± 0.17
	IM + 0.25 CAY10683	1.0362 ± 0.08**
	IM + 0.5 CAY10683	0.6237 ± 0.11**

a The results are expressed as the mean ± SEM. *n* = 3. **P < 0.01 *versus* the IM group.

**Table 3:** The characteristics of CML patients^a^

Samples	Age	Sex	CML phase	Imatinib resistant	WBC (10^9^ L)	Hb (g L^−1^)	PLT (10^9^ L)	Cytogenetics	% blasts (BM)	Mutations
CML1	34	M	BP	Yes	270.42	60	122	t(9,22)	30.26	Not
CML2	42	M	BP	Yes	15.34	86	310	t(9,22)	34.53	Not
CML3	39	F	BP	Yes	13.76	50	47	t(9,22)	45.29	Not

a Abbreviation: M: male; F: female; CML: chronic myeloid leukemia; BP: blast crisis phase; WBC: white blood cell; Hb: hemoglobin; PLT: platelets; BM: bone marrow.

Peripheral blood cells were obtained from healthy donors and IM-resistant CML patients. MNCs were isolated using Ficoll Lymphoprep (Axis-Shield PoCAs, Oslo, Norway) density gradient centrifugation. Then CD34^+^ progenitor cells were isolated by magnetic-assisted cell sorting (Miltenyi Biotech, Auburn, CA), and the purity was checked with anti-CD34-PE (BD Biosciences, San Jose, CA).

### 2.5 Reagents and antibodies

Novel selective HDAC2 inhibitor CAY10683, IM (STI571) and LY294002 were purchased from Selleck Chemicals (USA). Annexin V-fluorescein isothiocyanate/PI apoptosis detection kit was obtained from BD Biosciences (BD Biosciences, San Jose, CA, USA). Antibodies specific for C-caspases3, C-poly (ADP-ribose) polymerase (PARP), P21, CDK1, β-actin, HDAC2, P-PI3K, PI3K, P-AKT, AKT were bought from Santa Cruz Biotechnology (Santa Cruz, CA). Secondary antibodies (HRP-conjugated goat anti-rabbit or anti-mouse) were also purchased from Santa Cruz Biotechnology (Santa Cruz, CA).

### 2.6 Cell counting kit-8 (CCK-8) proliferation assay

Cells were seeded into 96-well plates at a density of 5 × 10^3^ cells per well with 3 replicate wells of each condition. After incubation overnight, the cells were treated with different concentrations of CAY10683 and IM, alone or in combination, for 48 h. For the CCK-8 assay, 10 μL of CCK-8 solution (Dojindo, Kumamoto, Japan) was added to each well and incubated at 37 °C for 2 h. Absorbance values at 450 nm were measured using a spectrophotometer (Molecular Devices, Sunnyvale, California, USA).

### 2.7 Calculation of CI

The combination index (CI) values were calculated by using the IC_50_ values of the treated cell lines and the computer software CalcuSyn (version 2.0). CI < 1, CI = 1, or CI > 1 correspond to the synergism, additivity, or antagonism of the applied substances.

### 2.8 Annexin V-FITC/propidium iodide (PI) staining for apoptosis analysis

Each treated cell was washed with PBS and stained with 5 μL of Annexin V-FITC at room temperature for 15 min in the dark and then with 10 μL of propidium iodide (PI) at 4 °C for 5 min in the dark. Apoptotic cells were detected using flow cytometry (BD Biosciences, San Jose, CA, USA), and the data were analyzed by Cell Quest software (BD Biosciences).

### 2.9 Cell cycle analysis

Cells were harvested, washed once in ice-cold PBS, fixed in 70% ethanol at 4 °C for more than 2 h, then incubated in a staining cocktail containing 50 μg mL^−1^ PI and 50 μg mL^−1^ RNase (BD Biosciences, San Jose, CA, USA) for 30 min at 37 °C in the dark. The DNA contents of the samples were analyzed using FACSCalibur flow cytometer (BD Biosciences) and Multicycle software.

### 2.10 Western blot analysis

Protein lysate was extracted from cells using RIPA lysis buffer supplemented with 1 μM PMSF (Solarbio Science & Technology, Beijing, China) agitated at 4 °C for 30 min, followed by centrifugation for 10 min. Supernatants were then loaded on 10% SDS–PAGE gel and the separated proteins were transferred onto PVDF membranes (EMD Millipore, Bedford, MA, USA) that were routinely blocked in 5% nonfat milk in PBS for 2 h with agitation and washed, then the membrane was blotted with primary antibodies for 2 h. After washing, the membranes were incubated with secondary antibodies (HRP-conjugated goat anti-rabbit or anti-mouse; Santa Cruz Biotechnology, Santa Cruz, CA) for 45 min at room temperature. All protein bands were detected using a Tanon 4200 automatic chemiluminescence image analysis system (Tanon, Shanghai, China). The protein bands were quantified by the integration of the chemiluminescence signals using Quantity One software (Bio-Rad Laboratories, Hercules, CA).

### 2.11 Construction of recombinant lentiviral vectors and transfection

Self-prepared recombinant lentivirus-V5-D-TOPO-HDAC2-EGFP and control vector lentivirus-V5-D-TOPO-EGFP were co-transfected into the 293FT packaging cell line. The supernatant was collected 48 h after transfection to harvest the recombinant virus. Lentivirus-V5-D-TOPO-EGFP-HADC2 and its empty vector were co-transfected into LAMA84-R and K562-R cells. In addition, the positivity of lentivirus-mediated HDAC2 transduction was observed by fluorescence microscopy, and the transfection efficiency was detected by the western blot method.

### 2.12 Xenograft mouse model of CML

Female 5 to 6 week-old non-obese diabetic severely compromised immunodeficient (NOD/SCID) mice were obtained from Beijing HEK Bioscience. K562-R cells (1 × 10^7^) were implanted with matrigel (BD Biosciences) subcutaneously into the right flank of sub-lethally irradiated (250 cGy) NOD/SCID mice. All mice were randomized into two groups, namely, a vehicle control group and a treatment group (*n* = 4 per group). Mice were treated with CAY10683 (50 mg kg^−1^; intraperitoneally), IM (50 mg kg^−1^; intraperitoneally), or with both agents daily for 21 days. Tumor volume was measured twice per week with calipers and was calculated as tumor volume (mm^3^) = *L* × *W*^2^/2 (*L* represents the largest diameter and *W* is the smallest diameter of the tumor). The weights of the mice were also monitored. The survival times of the mice were recorded and analyzed. All animal experiments were approved by the Ethics Committee of Guizhou Medical University.

### 2.13 HE staining

Fresh mice tissues were put into the stationary solution (4% formalin). The cell protein was denatured and solidified. The tissue was fixed for 24 h. After pruning the tissue, we put it into an embedding box and rinsed it with water for 30 min. Different concentrations of alcohol were used to dehydrate tissue blocks. Finally, the tissue blocks were placed in xylene. The transparent tissue blocks were placed in dissolved paraffin wax and stored in a wax box. After the tissue was completely immersed in the paraffin wax, paraffin was embedded. After the block was cooled and solidified, we used a slicing machine to slice the block. The slices were stained with hematoxylin solution for several minutes. The slices were placed in acid and ammonia water for a few seconds respectively. After 1 h of washing, the slices were placed in the distilled water for a moment, dehydrated in alcohol for 10 min, and stained for 2 to 3 min. The stained sections were dehydrated with pure alcohol and then made translucent by xylene and sealed with a cover glass. All experiments were conducted at least three times.

### 2.14 Terminal deoxynucleotidyl transferase dUTP nick end labeling (TUNEL) assay

Cellular apoptosis was assessed using the standard *in situ* cell death detection kit (Roche Diagnostics, Indianapolis, IN, USA) according to the manufacturer's instructions as previously described.^21^ In brief, the tissue sections were deparaffinized and rehydrated through a series of xylene and ethanol solutions. Permeabilization of tissue was carried out with proteinase K (Sigma-Aldrich, St. Louis, MO, USA) in 10 mM Tris 7.5 and 5 mM EDTA. After washing with PBS, the sections were incubated with TUNEL dye at RT for stipulated time intervals under dark conditions, whereas the nucleus was counter-stained with DAPI. Following staining, images were acquired on the fluorescence microscope (Olympus Microscope CKX53, Tokyo, Japan) in a detection range of 515–565 nm.

### 2.15 Immunohistochemistry

Expression of HDAC2 was performed by immunohistochemistry staining as previously described.^22^ About 5 μm thick paraffin sections were cut, deparaffinized, and rehydrated. After endogenous peroxidase activity was blocked, the sections were incubated with HDAC2 primary antibody at 4 °C overnight. Finally, staining was carried out using 3′-diaminobenzidine (DAB; Bioss, China). Five visual fields were randomly selected (cell number, ≥200).

### 2.16 Statistical analysis

Each experiment was repeated at least three times and the most representative example was chosen. Statistical analysis of the experimental data was performed by using GraphPad Prism 5 software (GraphPad Software Inc, San Diego, CA). Data are reported as mean ± SEM. Statistically significant differences between the treatment groups were calculated using Student's *t*-test. Differences are considered statistically significant at *p* values <0.05.

## 3 Results

### 3.1 CAY10683 combined with IM exerted a synergistic effect on inhibiting the viability of CML cells resistant to IM

**Fig. 1:**
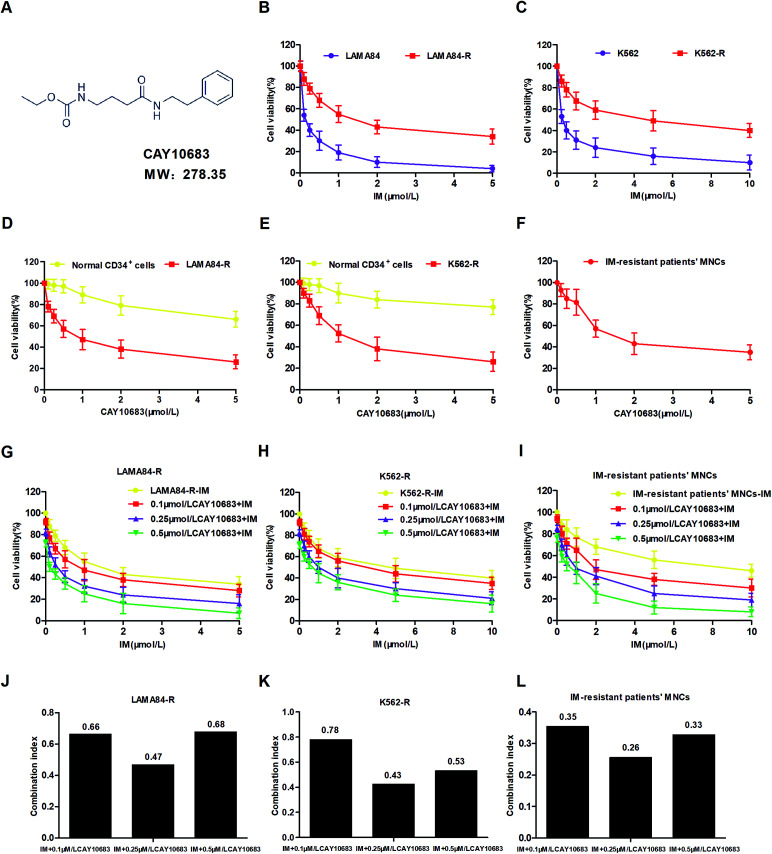
CAY10683 combined with IM exerted a synergistic effect in inhibiting the viability of CML cells resistant to IM. (A) Chemical structure of CAY10683. (B) LAMA84-R and LAMA84 cells were subjected to 48 h of IM treatments at various doses, and the CCK-8 assay was conducted to determine the cell viability. (C) K562-R and K562 cells were subjected to 48 h of IM treatments at various doses, and the CCK-8 assay was conducted to determine the cell viability. (D) Normal CD34^+^ and LAMA84-R cells were subjected to 48 h of CAY10683 treatments at various doses, and the CCK-8 assay was conducted to determine the cell viability. (E) Normal CD34^+^ and K562-R cells were subjected to 48 h of CAY10683 treatments at various doses, and the CCK-8 assay was conducted to determine the cell viability. (F) IM-resistant CML patients' MNCs were subjected to 48 h of CAY10683 treatments at various doses, and the CCK-8 assay was conducted to determine the cell viability. (G) LAMA84-R cells were subjected to 48 h of IM (0–5 μM) and CAY10683 (0, 0.1, 0.25, 0.5 μM) treatment, and the CCK-8 assay was conducted to determine the cell viability. (H) K562-R cells were subjected to 48 h of IM (0–10 μM) and CAY10683 (0, 0.1, 0.25, 0.5 μM) treatment, and the CCK-8 assay was conducted to determine the cell viability. (I) IM-resistant CML patients' MNCs were subjected to 48 h of IM (0–10 μM) and CAY10683 (0, 0.1, 0.25, 0.5 μM) treatment, and the CCK-8 assay was conducted to determine the cell viability. (J) The CI values of IM and CAY10683 within LAMA84-R cells were determined using the CalcuSyn software (version 2.0). (K) The CI values of IM and CAY10683 within K562-R cells were determined by the CalcuSyn software (version 2.0). (L) The CI values of IM and CAY10683 within IM-resistant CML patients' MNCs were determined by the CalcuSyn software (version 2.0). CI < 1 indicates the synergistic effect; CI = 1 represents the additive effect; CI > 1 suggests the antagonistic effect. All experiments were conducted three times. All results are presented in the form of mean ± SEM. *n* = 3. *P < 0.05, **P < 0.01 and ***P < 0.001.

To examine the IM resistance of K562-R and LAMA84-R cells, the IC_50_ values of single IM treatment were evaluated in the resistant and sensitive CML cells by the CCK-8 assay after incubation for 48 h. The K562 and LAMA84 cells sensitive to IM showed high susceptibility to IM alone, which was verified by the IC_50_ values of 0.2847 and 0.1411 μM, respectively (Tables 1 and 2). Typically, the resistance values of K562-R and LAMA84-R cells were about 16.02 and 11.03 times higher as compared to those of K562 and LAMA84 cells sensitive to IM, respectively (Fig. 1B and C). Forty-eight hours of CAY10683 treatment inhibited K562-R, LAMA84-R and IM-resistant CML patients' MNC cell proliferation in a dose-dependent manner. Nonetheless, CAY10683 at identical doses only resulted in minimal effects on normal CD34^+^ cells, which indicated negligible drug cytotoxicity to normal cells (Fig. 1D–F). To further examine the CAY10683 combined with IM inhibition on CML cells resistant to IM, K562-R, LAMA84-R and IM-resistant CML patients' MNC cells were subjected to 48 h of CAY10683 (0, 0.1, 0.25, 0.5 μM, CAY10683 at 0–0.5 μM had little effect on the normal CD34^+^ cells) and IM treatments at various doses. Afterwards, the CCK8 assay was conducted to determine the viability of the cells. According to Fig. 1G–I, the combined treatment boosted the antiproliferative effects on K562-R, LAMA84-R and IM-resistant CML patients' MNC cells in comparison with those in the respective monotherapies. Tables 1, 2 and 4 display the IC_50_ values for IM combined with CAY10683 at various doses, together with the corresponding statistical differences. The CI value was determined on the basis of the IC_50_ value, which was used to verify the synergistic effect of these two drugs. In these three cell lines, 0.25 μM CAY10683 combined with IM achieved the most obvious synergistic effect (Fig. 1J–L). Therefore, 0.25 μM CAY10683 was selected for subsequent experiments.

**Table 4:** Sensitivity of imatinib-resistant patients' MNCs to IM, CAY10683 or their combination^a^

Cell lines	IM + CAY10683 (μM)	IC_50_ value (μM)
IM-resistant patients' MNCs	IM	7.5315 ± 0.51
	CAY10683	1.9615 ± 0.77
	IM + 0.1 CAY10683	2.2835 ± 0.56***
	IM + 0.25 CAY10683	0.9675 ± 0.12***
	IM + 0.5 CAY10683	0.5500 ± 0.19***

a The results are expressed as the mean ± SEM. *n* = 3. ***P < 0.001 *versus* the IM group.

### 3.2 CAY10683 combined with IM exerted synergistic effects on inducing the apoptosis of CML cells resistant to IM

**Fig. 2:**
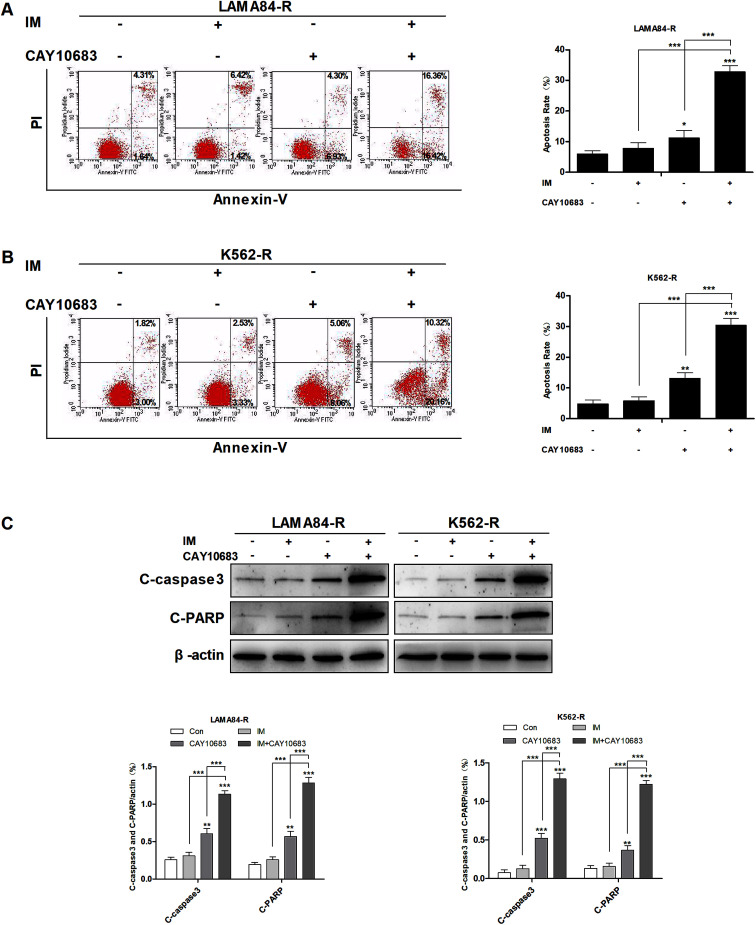
CAY10683 combined with IM exerted a synergistic effect on inducing the apoptosis of CML cells resistant to IM. (A) LAMA84-R cells were subjected to 48 h of CAY10683 (0.25 μM) and IM (0.5 μM) treatment alone, or the combination of these two, and flow cytometry was conducted to measure the apoptotic cells. (B) K562-R cells were subjected to 48 h of CAY10683 (0.25 μM) and IM (1 μM) treatment alone, or the combination of these two, and flow cytometry was conducted to measure the apoptotic cells. (C) Western blotting was conducted to determine the expression of apoptosis-associated proteins in K562-R and LAMA84-R cells. All experiments were conducted three times. All results are presented in the form of mean ± SEM. *n* = 3. *P < 0.05, **P < 0.01 and ***P < 0.001.

To examine the synergy of CAY10683 with IM on inducing apoptosis, LAMA84-R cells were subjected to 48 h of CAY10683 (0.25 μM) and IM (0.5 μM) treatments, and the combination of these two, whereas the K562-R cells were subjected to 48 h of CAY10683 (0.25 μM) and IM (1 μM) treatments, and the combination of these two. Then, the apoptotic cells were detected through flow cytometry. According to our findings, the apoptotic rate in cells treated with IM for 48 h was almost the same as that in cells without IM treatment, while 0.25 μM CAY10683 resulted in the obvious apoptosis of K562-R and LAMA84-R cells. It was interesting that the combined treatment resulted in markedly elevated apoptotic rates of K562-R and LAMA84-R cells in comparison with those in respective monotherapy (Fig. 2A and B). Later, western blotting was carried out to detect the influence of a single drug treatment or combined treatment on apoptosis-related protein expression. According to Fig. 2C, C-PARP and C-caspase3 (proteolytic cleavages of PARP and caspase3) protein expressions were markedly up-regulated in K562-R and LAMA84-R cells of the combined treatment group in comparison with that in the respective monotherapy groups. The above findings potently support that CAY10683 combined with IM synergistically and effectively boosted apoptosis of the CML cells resistant to IM relative to the respective monotherapy; *i.e.*, the CAY10683 combined with IM treatment reversed the CML resistance to IM.

### 3.3 CAY10683 combined with IM exerted a synergistic effect on inducing cell cycle arrest at the G2/M phase in CML cells resistant to IM

**Fig. 3:**
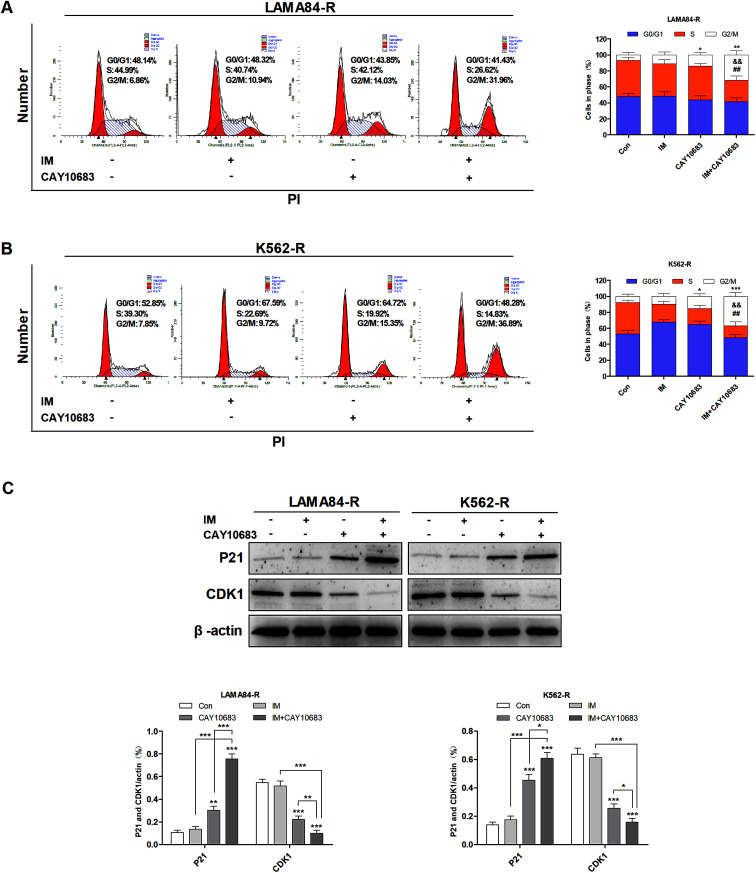
CAY10683 combined with IM exerted a synergistic effect on inducing cell cycle arrest in the G2/M phase in CML cells resistant to IM. (A) LAMA84-R cells were subjected to 24 h of CAY10683 (0.25 μM) and IM (0.5 μM) treatment alone, or the combination of these two, and flow cytometry was conducted to determine the cell cycle distribution. (B) K562-R cells were subjected to 24 h of CAY10683 (0.25 μM) and IM (1 μM) treatment alone, or the combination of these two, and flow cytometry was conducted to determine the cell cycle distribution. (C) Western blotting was carried out to determine the cell cycle-associated protein levels in K562-R and LAMA84-R cells. All experiments were conducted three times. All results are presented in the form of mean ± SEM. *n* = 3. *P < 0.05, **P < 0.01 and ***P < 0.001, &&*P* < 0.01 *versus* IM group, ##*P* < 0.01 *versus* CAY10683 group.

To examine the influence of CAY10683 combined with IM on regulating the cell cycle distribution, LAMA84-R cells were subjected to 24 h of CAY10683 (0.25 μM) and IM (0.5 μM) treatment, or CAY10683 combined with IM. Besides, K562-R cells were subjected to 24 h of CAY10683 (0.25 μM) and IM (1 μM) treatment, or CAY10683 combined with IM, then flow cytometry was used to analyze the cell cycle distribution. According to Fig. 3A and B, the flow cytometry results suggest that IM made no difference in the cell cycle distribution in K562-R and LAMA84-R cells, whereas CAY10683 blocked the cell cycle arrest in the G2/M phase. Additionally, the combined treatment markedly boosted cell cycle arrest in the G2/M phase of K562-R and LAMA84-R cells in comparison to the respective monotherapies. To shed light on the underlying mechanism of cell cycle arrest, the cell cycle-related protein expression was detected through western blotting. Our results suggest that CDK1 was down-regulated following combined treatment, whereas P21 was up-regulated (Fig. 3C). The above findings revealed that CAY10683 combined with IM exerted a synergistic effect on inducing cell cycle arrest (G2/M) of CML cells resistant to IM.

### 3.4 CAY10683 combined with IM resulted in apoptosis of CML cells resistant to IM mainly through inhibiting HDAC2

**Fig. 4:**
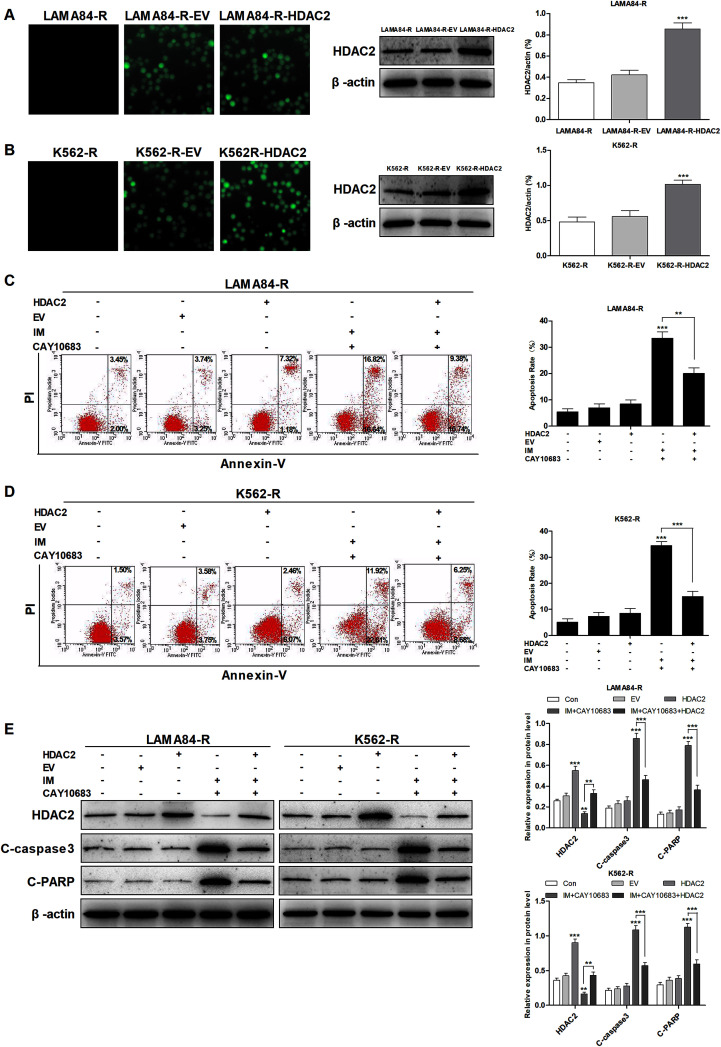
CAY10683 combined with IM induced the apoptosis of CML cells resistant to IM mainly through suppressing HDAC2. (A) Fluorescence microscopy was conducted to observe the positiveness of lentivirus-transfected HDAC2 within LAMA84-R cells, whereas western blotting was performed to determine HDAC2 expression. (B) Fluorescence microscopy was conducted to observe the positiveness of lentivirus-transfected HDAC2 within K562-R cells, whereas western blotting was performed to determine HDAC2 expression. (C) The apoptotic rates in LAMA84-R-HDAC2 and LAMA84-R-Con cells under 48 h of CAY10683 (0.25 μM) combined with IM (0.5 μM) treatment were evaluated through flow cytometry. (D) The apoptotic rates in K562-R-HDAC2 and K562-R-Con cells under 48 h of CAY10683 (0.25 μM) combined with IM (1 μM) treatment were evaluated through flow cytometry. (E) Western blotting was carried out to determine the expression of HDAC2 as well as apoptosis-associated proteins in K562-R and LAMA84-R cells. All experiments were conducted three times. All results are presented in the form of mean ± SEM. *n* = 3. *P < 0.05, **P < 0.01 and ***P < 0.001.

As suggested in previous studies, the over-expression of HDACs is an obvious mechanism that results in the drug resistance of leukemia.^16,17^ Furthermore, HDAC2 has been reported to be highly expressed in K562-R cells resistant to IM as compared to K562 cells sensitive to IM, indicating that HDAC2 may play a critical role in developing IM resistance.^20^ Previous studies suggest that CAY10683 combined with IM treatment reverses the IM resistance of CML. Therefore, this study investigated whether the effect of CAY10683 (a selective HDAC2 inhibitor) combined with IM on CML cells resistant to IM was mainly achieved through inhibiting HDAC2. HDAC2 was up-regulated in K562-R and LAMA84-R cells through lentiviral transfection, and fluorescence microscopy was used to detect EGFP. The results of western blotting suggested that HDAC2 showed high expression in K562-R-HDAC2 and LAMA84-R-HDAC2 cells relative to that in K562-R-EV and LAMA84-R-EV cells, respectively (Fig. 4A and B). Following the combined treatment with CAY10683 and IM, the protein levels of HDAC2 in K562-R-HDAC2 and LAMA84-R-HDAC2 cells were markedly higher as compared to those in K562-R-Con and LAMA84-R-Con cells, respectively (Fig. 4E). Following the combined treatment with CAY10683 and IM, apoptosis rates in K562-R-HDAC2 and LAMA84-R-HDAC2 cells were markedly lower as compared to those in K562-R-Con and LAMA84-R-Con cells, respectively (Fig. 4C and D). The above findings suggest that HDAC2 up-regulation protected CML cells resistant to IM from apoptosis induced by the combined treatment. As a result, anti-apoptotic signal transduction pathways participating in HDAC2 up-regulation were studied at the protein level (C-PARP and C-caspase3). Accordingly, HDAC2 transfection reduced the activation of PARP and caspase3 resulting from the combined treatment (Fig. 4E). The data suggest that HDAC2 up-regulation in CML cells resistant to IM partially recovered the apoptosis-associated protein expression in combined treatment. Taken together, CAY10683 combined with IM resulted in apoptosis of CML cells resistant to IM mainly through inhibiting HDAC2.

### 3.5 CAY10683 combined with IM induced cell cycle arrest at the G2/M phase of CML cells resistant to IM mainly through inhibiting HDAC2

**Fig. 5:**
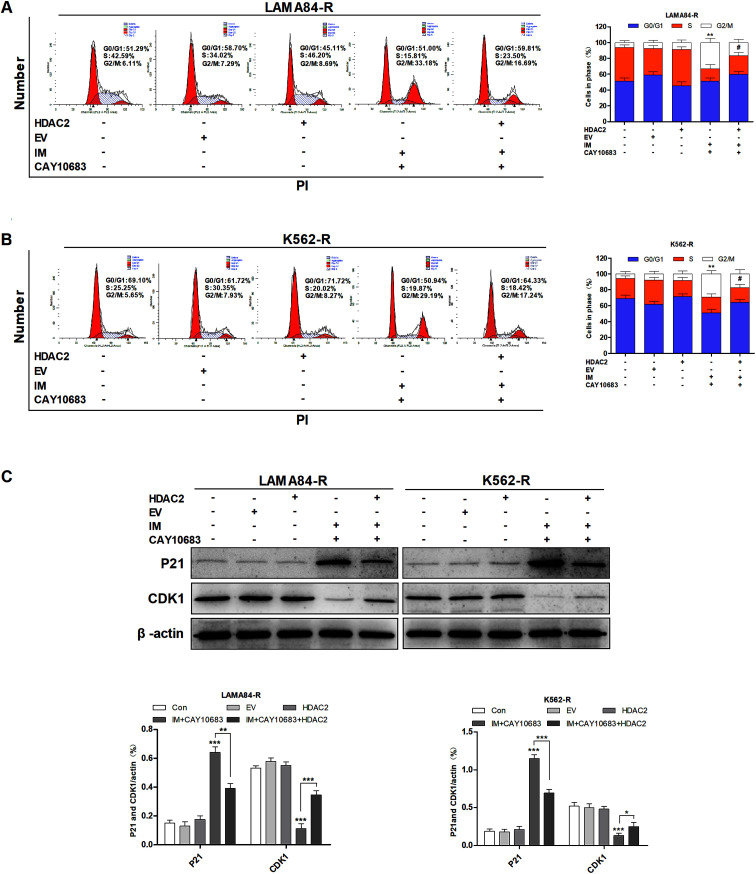
CAY10683 combined with IM treatment induced cell cycle arrest in the G2/M stage in CML cells resistant to IM mainly through inhibiting HDAC2. (A) Flow cytometry was employed to assess the cell cycle distribution in LAMA84-R-HDAC2 and LAMA84-R-Con cells under 24 h of combined treatment with CAY10683 (0.25 μM) and IM (0.5 μM). (B) Flow cytometry was employed to assess the cell cycle distribution in K562-R-HDAC2 and K562-R-Con cells under 24 h of combined treatment with CAY10683 (0.25 μM) and IM (1 μM). (C) Western blotting was conducted to determine the expression of cell cycle-related proteins in K562-R and LAMA84-R cells. All experiments were conducted three times. All results are presented in the form of mean ± SEM. *n* = 3. *P < 0.05, **P < 0.01 and ***P < 0.001.

According to previous experiments, CAY10683 combined with IM exerted a synergistic effect on inducing the cell cycle arrest in the G2/M phase in CML cells resistant to IM. To examine the underlying mechanism regarding the synergy of CAY10683 combined with IM in the cell cycle distribution of CML cells resistant to IM, it was hypothesized that HDAC2 over-expression might reduce the cell cycle arrest in the G2/M phase of CML cells resistant to IM. After combined treatment with CAY10683 and IM, fewer cells in K562-R-HDAC2 and LAMA84-R-HDAC2 groups were arrested in the G2/M phase as compared to those in K562-R-Con and LAMA84-R-Con groups, respectively (Fig. 5A and B). These findings indicate that HDAC2 up-regulation protected CML cells resistant to IM from cell cycle arrest in the G2/M phase induced by the combined treatment. To validate the above findings, the protein expressions of cell cycle-associated proteins (CDK1 and P21) were determined. After combined treatment, P21 expression in K562-R-HDAC2 and LAMA84-R-HDAC2 cells was down-regulated, whereas CDK1 was up-regulated relative to that in K562-R-Con and LAMA84-R-Con cells, respectively (Fig. 5C). These findings suggest that over-expression of HDAC2 partially reversed the cell cycle-associated protein expression caused by CAY10603 combined with IM treatment. Taken together, the above findings potently indicate that CAY10683 combined with IM treatment induced cell cycle arrest in the G2/M phase of CML cells resistant to IM mainly through inhibiting HDAC2.

### 3.6 CAY10683 combined with IM caused apoptosis of CML cells resistant to IM partly through the HDAC2-mediated PI3K/Akt signaling pathway

**Fig. 6:**
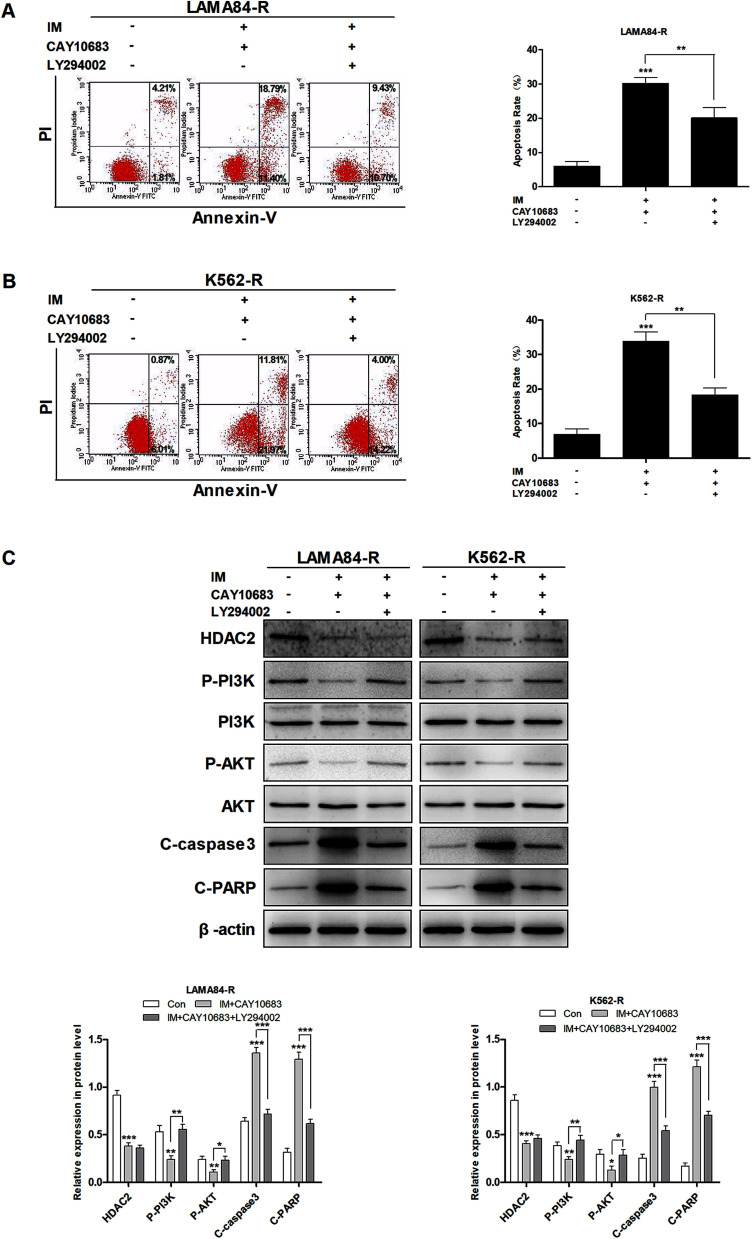
CAY10683 combined with IM treatment induces the apoptosis of CML cells resistant to IM partially through the HDAC2-regulated PI3K/Akt signal transduction pathway. (A) LAMA84-R cells are subjected to 12 h of LY294002 (20 nM) treatment, followed by the addition of CAY10683 (0.25 μM) and IM (0.5 μM). Cell apoptosis was determined after 48 h using the Annexin V-FITC and PI staining kit. (B) K562-R cells are subjected to 12 h of LY294002 (20 nM) treatment, followed by the addition of CAY10683 (0.25 μM) and IM (1 μM). Cell apoptosis was determined after 48 h using the Annexin V-FITC and PI staining kit. (C) Associated protein levels are assessed through western blotting. All experiments were conducted three times. All results are presented in the form of mean ± SEM. *n* = 3. *P < 0.05, **P < 0.01 and ***P < 0.001.

**Fig. 7:**
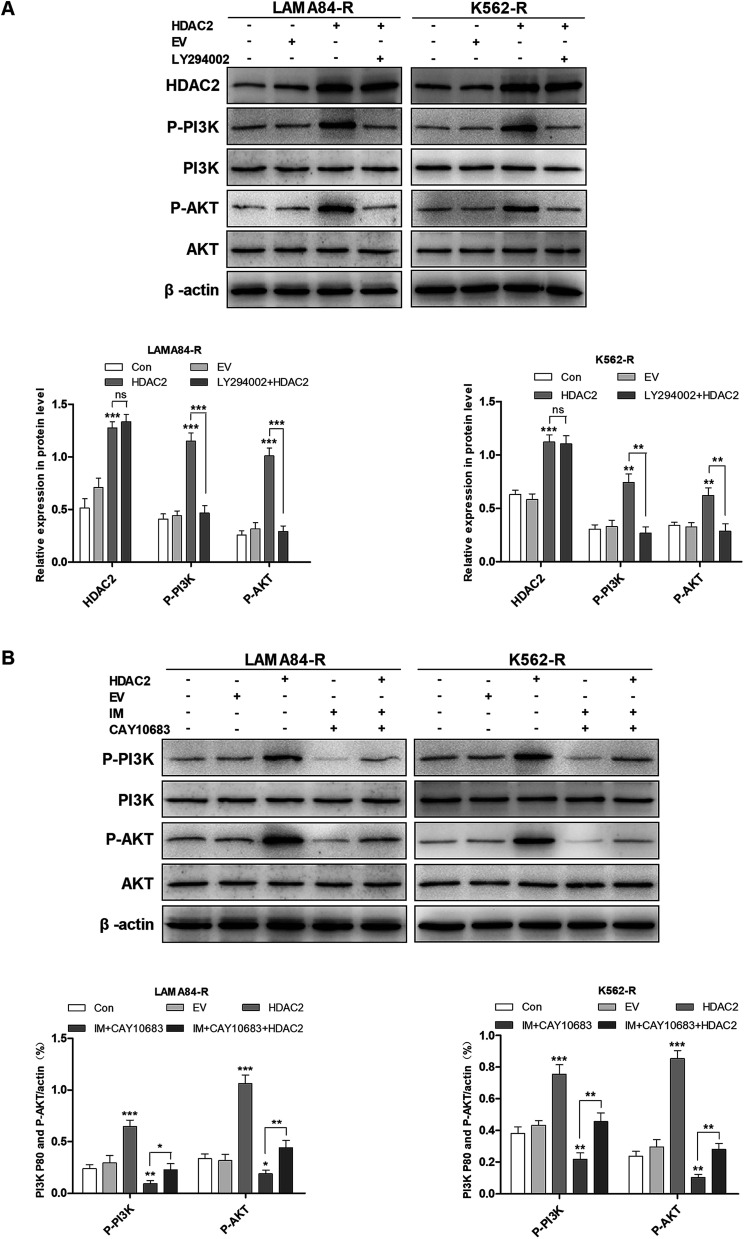
The PI3K/Akt signal transduction pathway mediated HDAC2 regulation of CML cells resistant to IM. (A) According to the results of western blotting, HDAC2 up-regulation mediated the phosphorylation of PI3K/Akt in CML cells resistant to IM, while the over-expression of HDAC2 was not blocked following 12 h treatment with 20 nM LY294002. (B) Western blotting was carried out to detect the PI3K/Akt pathway-associated protein expression in LAMA84-R-HDAC2 and LAMA84-R-Con cells under combined treatment with CAY10683 (0.25 μM) and IM (0.5 μM) for 48 h. Western blotting was conducted to detect the PI3K/Akt pathway-associated protein expression in K562-R-HDAC2 and K562-R-Con cells under combined treatment with CAY10683 (0.25 μM) and IM (1 μM) for 48 h. All experiments were conducted three times. All results are presented in the form of mean ± SEM. *n* = 3. *P < 0.05, **P < 0.01 and ***P < 0.001.

It was mentioned previously that the treatment combining CAY10683 with IM resulted in apoptosis of CML cells resistant to IM mainly through inhibiting HDAC2; nevertheless, the pathway through which over-expression of HDAC2 modulated the apoptosis of CML cells resistant to IM is unknown. The PI3K/Akt signal transduction pathway was verified to be a crucial factor associated with cell apoptosis, differentiation and proliferation.^23^ The abnormally activated PI3K/Akt signal transduction pathway is reported in many cancers, which usually results from the overexpression of Akt or PI3K. Besides, this pathway is a downstream signal that is activated *via* the BCR–ABL kinase, which is suggested to play an indispensable role in CML cell survival mediated by BCR–ABL.^24^ Therefore, it was postulated in this study that the PI3K/Akt signal transduction pathway participated in the association of HDAC2 with the apoptosis of CML cells resistant to IM. Subsequently, cells were subjected to CAY10683 combined with IM treatment with or without LY294002, the inhibitor of the PI3K/Akt pathway. As shown in Fig. 6A and B, LY294002 markedly attenuated the apoptosis induced by combined therapy in K562-R and LAMA84-R cells. Besides, immunoblotting results revealed that LY294002 blocked the inhibition of the PI3K/Akt pathway, as well as the changes in apoptosis-associated proteins (like C-PARP and C-caspase3) induced by the combined treatment. Nonetheless, LY294002 did not block HDAC2 inhibition resulting from the combined treatment (Fig. 6C), suggesting that HDAC2 inhibition resulting from the combined treatment was an event that occurred earlier than PI3K/Akt suppression and cell apoptosis. After up-regulating HDAC2 expression in both K562-R and LAMA84-R cells, LY294002 suppressed the PI3K/Akt signal pathway. Afterwards, western blotting was carried out to detect the expression of HDAC2 as well as the PI3K/Akt pathway-associated proteins (PI3K, P-PI3K, AKT, and P-AKT) following 12 h of 20 nM LY294002 treatment. According to Fig. 7A, western blotting revealed that the up-regulation of HDAC2 mediated the phosphorylation of PI3K/Akt in both K562-R and LAMA84-R cells. LY294002 evidently reduced the phosphorylation of PI3K/Akt in CML cells resistant to IM with up-regulated HDAC2, while the over-expression of HDAC2 was not blocked through LY294002. The above findings suggest that the regulation of HDAC2 affected the downstream PI3K/Akt signal transduction pathway in CML cells resistant to IM. P-AKT and P-PI3K expression at the protein level was dramatically up-regulated in K562-R-HDAC2 and LAMA84-R-HDAC2 cells following combined treatment with CAY10683 and IM relative to that in K562-R-Con and LAMA84-R-Con cells, respectively (Fig. 7B). The data suggest that HDAC2 up-regulation protected CML cells resistant to IM from the inhibition of the PI3K/Akt pathway resulting from the combined treatment. Collectively, our results reveal that the CAY10683 combined with IM treatment induced apoptosis of CML cells resistant to IM partially through the PI3K/Akt pathway mediated by HDAC2.

### 3.7 CAY10683 combined with IM exerted synergistic effects on inhibiting CML proliferation *in vivo*

**Fig. 8:**
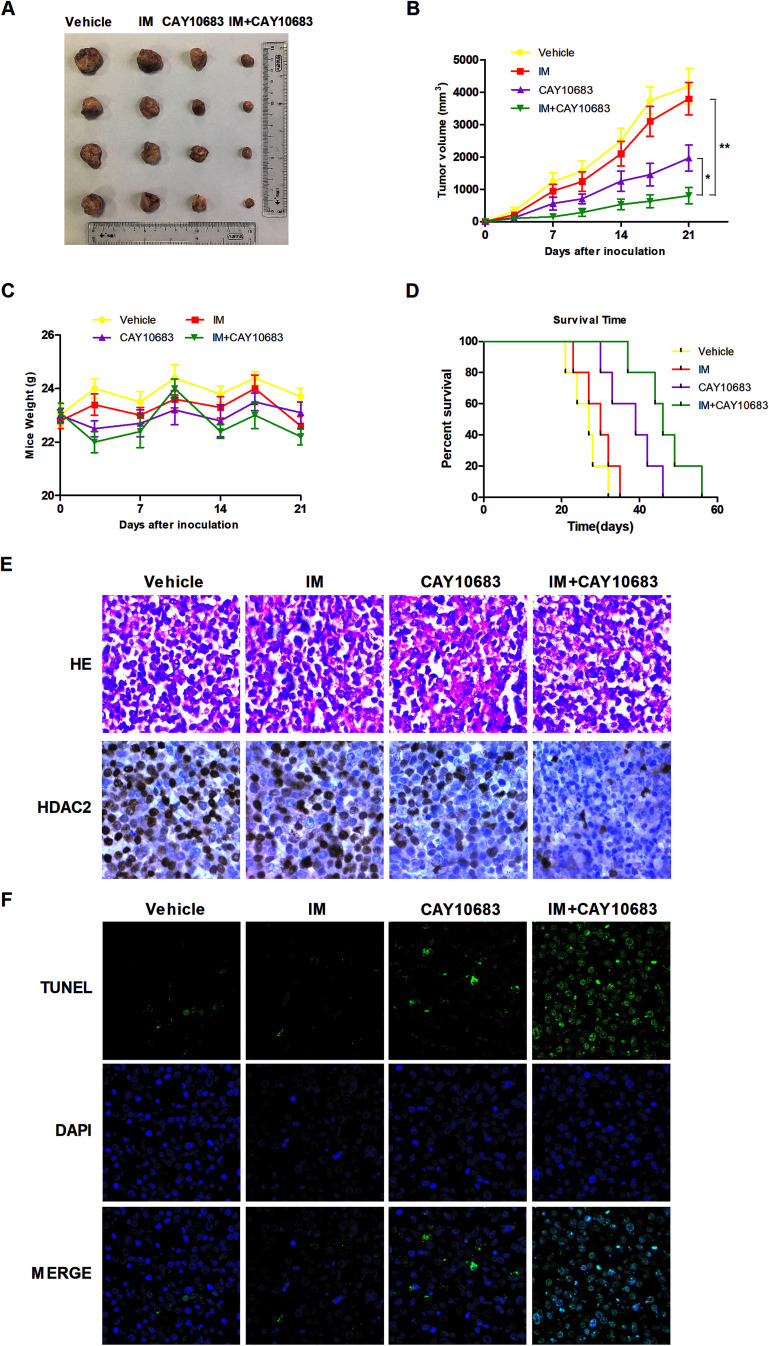
CAY10683 combined with IM exerted a synergistic effect on inhibiting CML proliferation *in vivo*. (A) Images for tumors were collected based on the NOD/SCID mice in each group. (B) IM, CAY10683, or the combination of these two, were given intraperitoneally once per day to NOD/SCID mice that were subcutaneously implanted with the K562-R cells. The caliper was used to measure tumor size on the 0th, 3rd, 7th, 10th, 14th, 17th, and 21st days, respectively. The calculated values are depicted in the Materials and Methods section. (C) The weights of the mice were assessed for all groups. (D) The survival curves for all groups were estimated from treatment initiation to death by the use of the Kaplan–Meier curves. (E) The H&E staining for xenografts in each group and HDAC2 protein expression in mouse xenograft tissues of every group were evaluated through immunohistochemistry. (F) Typical TUNEL staining images. Fluorescence microscopy was employed for identifying apoptotic cells labeled by TUNEL (green), and the cell nuclei stained with DAPI (blue). Cells displaying green fluorescence were recognized to be the TUNEL positive cells. All experiments were conducted three times. All results are presented in the form of mean ± SEM. *n* = 3. *P < 0.05, **P < 0.01.

According to previous experiments, CAY10683 combined with IM had synergy in CML cells resistant to IM mainly *via* inhibiting HDAC2. In the next experiment, the synergistic effects *in vivo* were investigated by the xenograft mouse model. To this end, K562-R cells were subcutaneously injected into the right flanks in the SCID mice. All animals were then randomly divided into the treatment group (*n* = 4) and vehicle control (*n* = 4) group. The animals were subjected to 50 mg kg^−1^ CAY10683 (intraperitoneal injection) and 50 mg kg^−1^ IM (intraperitoneal injection) treatment, or the combination of these two for 21 consecutive days. Our results suggested that CAY10683 combined with IM treatment had a remarkable antitumor effect on the K562-R xenograft model in comparison with IM or CAY10683 treatment alone. Besides, IM and/or CAY10683 treatment resulted in no obvious weight loss or toxicity (Fig. 8A–C). Meanwhile, the overall survival (OS) was found to be markedly extended in the CML mouse model subjected to CAY10683 combined with IM treatment relative to the respective monotherapy (*p* < 0.05; Fig. 8D). H&E staining of xenografts revealed the irregular and disordered arrangement of tumor cells with elevated nucleus-cytoplasm ratio, which is in good agreement with the malignancy pathological characteristics. Besides, the combined treatment resulted in the dramatically down-regulated protein expression of HDAC2 in the xenograft mouse model in comparison with that in the respective monotherapies as evidenced by IHC (Fig. 8E). Interestingly, in comparison with the vehicle group or respective monotherapy group, the combined treatment group displayed an increased apoptotic cell percentage, as suggested by the elevated TUNEL-positive cells (Fig. 8F). The above results suggest that CAY10683 combined with IM treatment was well tolerated and exerted synergistic effects on inhibiting CML proliferation *in vivo*.

### 3.8 Potential underlying mechanism regarding the combined treatment-induced apoptosis

**Fig. 9:**
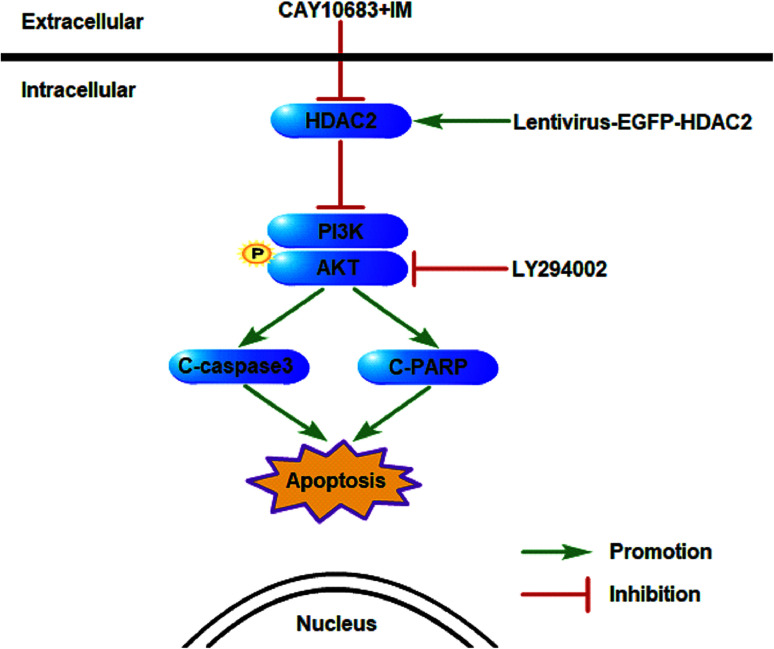
Schematic diagram regarding the underlying mechanisms of apoptosis resulting from CAY10683 combined with IM. The association between HDAC2 and the PI3K/Akt signaling pathway as well as the related downstream proteins following combined therapy are displayed.

The experiments carried out above suggest that the CAY10683 combined with IM treatment displayed synergistic effects mainly through inhibiting HDAC2. Additionally, the PI3K/Akt signaling pathway was also implicated in mediating HDAC2 regulation of apoptosis. Thus, it was postulated in this study that HDAC2 is a critical factor during this process. Fig. 9 shows the mechanism regarding the association of HDAC2 with the PI3K/Akt signaling pathway in apoptosis induced by combined treatment.

## 4 Discussion

Allo-SCT is currently recommended in existing treatment concepts for advanced CML following TKI pretreatment, as well as for cases that fail the TKI treatment, depending on the risk of transplantation.^25^ Failure in first-line IM treatment may lead to the occurrence of drug intolerance and resistance. Over the past ten years, increasing attention has been directed toward investigating multi-pronged targeted therapeutic strategies to manage the clinical problem of primary or evolving IM resistance.^26–28^ At present, more and more evidence indicates that combined treatment plays a vital role in handling IM resistance.

HDACs can regulate oncogene and tumor suppressor gene activities, which thereby play a vital part in tumorigenesis.^29^ HDACs are usually investigated in the preclinical studies on hematological malignancies and solid tumors such as the IM-resistant CML.^29,30^ In the current work, CAY10683, the new selective HDAC2 inhibitor, was utilized in combination with IM for treating hematological disorders for the first time. Our results indicate that the combined treatment had a synergistic effect on inhibiting cell viability while inducing apoptosis as well as cell cycle arrest at the G2/M phase in comparison with those of the respective monotherapies in CML cells resistant to IM; *i.e.*, CAY10683 combined with IM treatment reversed the CML resistance to IM. Such results are in agreement with prior reports regarding growth inhibition of HDACi plus TKIs on CML cells.^12^ Furthermore, it was suggested that CAY10683 combined with IM treatment had synergistic effects on the cells resistant to IM mainly through inhibiting HDAC2 and that the PI3K/Akt signal transduction pathway modulated HDAC2 regulation on CML cells resistant to IM. Eventually, it was discovered, based on the xenograft mouse model, that the combined treatment markedly suppressed CML proliferation *in vivo*. The above results highlighted that CAY10683 combined with IM had the potential to handle the CML resistance to IM.

Some therapeutics targeting IM resistance, including staurosporine,^31^ divalproex sodium,^12^ and panobinostat,^4^ were tested. Generally, it is suggested that IM resistance is related to BCR–ABL in CML.^32^ Nonetheless, numerous studies demonstrate that IM resistance is not associated with BCR–ABL in CML.^33^ Some researchers propose that the signal transduction pathways independent of BCR–ABL, such as HADCs, protein kinase C, and NF-κB transcription factor, participate in the resistance to IM in leukemia.^34–36^ Any change in HDACs is a crucial BCR–ABL-independent mechanism for the resistance to IM of CML cells.^36^ HDAC2, one of the class I HDACs members, may play a pathogenic role in leukemia.^37^ HDAC2 over-expression was found in CML cells resistant to IM.^20^ The above studies suggest that HDAC2 potentially plays a critical role in mediating the IM resistance independent of BCR–ABL. Also, our findings demonstrate that HDAC2 up-regulation protected CML cells resistant to IM from apoptosis as well as cell cycle arrest at the G2/M phase induced by the combined treatment. A recent study has indicated that the knockout of the HDAC2 gene can promote the apoptosis of K562 cells, which further indicated that HDAC2 up-regulation plays a crucial part in the maintenance of K562 cell survival.^37^ As such, HDAC2 plays a critical part in mediating IM resistance, independent of BCR–ABL. Consequently, IM resistance is not only associated with protein kinase C and NF-κB, but also with HDAC2 among CML cells.

According to prior research, the PI3K/Akt signal pathway is dysregulated in response to the activation of BCR–ABL, which is also modulated in response to IM treatment.^38^ Additionally, the inhibition of PI3K/Akt sensitizes the CML leukemic stem cells to TKIs.^39^ Liu *et al.* suggested that PI3K/Akt blocking within the bone marrow stromal cells dramatically reduced the IM resistance induced by HO-1.^40^ As shown in our study, the Akt signaling pathway had a negative effect on regulating apoptosis, so the HDAC2 effect on Akt activity in CML cells resistant to IM was also investigated. After HDAC2 up-regulation, PI3K and Akt phosphorylation was initiated. PI3K/Akt, the critical cell survival signaling in human beings, plays a role as the central pathway to regulate tumor cell survival.^41,42^ PI3K can be activated *via* numerous upstream cytokines and growth factors, which can thereby phosphorylate Akt. Akt activation potently modulates some downstream targets such as multidrug resistance protein 1 (MRP1), Bcl-2 family members, and caspase cascades.^43^ According to our findings, the apoptosis induced by combined treatment enhanced by HDAC2 suppression is correlated with suppressing the PI3K/Akt signaling pathway in CML cells resistant to IM, whereas PI3K/Akt suppression reversed such results. Nonetheless, the precise underlying mechanism regarding the association of HDAC2 with Akt in CML cells resistant to IM is still unknown. In addition, more studies are warranted to examine the influence of HDAC2 knockout in the mouse model. Nonetheless, CAY10683 combined with IM exerts synergistic effects, which potentially provide a therapeutic advantage for overcoming IM resistance, and more clinical studies with patient-derived samples are needed to validate its role.

## 5 Conclusion

This study is the first to illustrate the synergy of IM and CAY10683. Typically, the CAY10683 combined with IM treatment has been suggested to exert a synergistic effect on handling the IM resistance problem in CML, which is majorly achieved through inhibiting HDAC2. Taken together, the findings in this study shed new light on the impacts of combined treatment with CAY10683 (a selective HDAC) and IM, and lay a certain foundation for overcoming IM resistance.

## Conflicts of interest

The authors declare that they have no conflicts of interest.

## Supplementary Material
